# Sphingolipids in cardiovascular diseases and metabolic disorders

**DOI:** 10.1186/s12944-015-0053-y

**Published:** 2015-06-16

**Authors:** Sonia Borodzicz, Katarzyna Czarzasta, Marek Kuch, Agnieszka Cudnoch-Jedrzejewska

**Affiliations:** Department of Experimental and Clinical Physiology, Laboratory of Centre for Preclinical Research, First Faculty of Medicine, Medical University of Warsaw, Banacha 1b, 02-097 Warsaw, Poland; 1st Department of Cardiology, Medical University of Warsaw, Banacha 1a, 02-097 Warsaw, Poland; Department of Heart Failure and Cardiac Rehabilitation of the Chair and Department of Cardiology, Hypertension and Internal Diseases, Second Faculty of Medicine, Medical University of Warsaw, Kondratowicza 8, 03-242 Warsaw, Poland

**Keywords:** Sphingolipids, Ceramide, Sphingosine-1-phosphate, Cardiovascular diseases, Metabolic disorders

## Abstract

Many investigations suggest the pivotal role of sphingolipids in the pathogenesis of lifestyle diseases such as myocardial infarction, hypertension, stroke, diabetes mellitus type 2 and obesity. Some studies suggest that sphingolipids are important factors in cellular signal transduction. They serve as biologically active components of cell membrane and are involved in many processes such as proliferation, maturation and apoptosis. Recently, ceramide and sphingosine-1-phosphate have become the target of many investigations. Ceramide is generated in three metabolic pathways and many factors induce its production as a cellular stress response. Ceramide has proapoptotic properties and acts as a precursor for many other sphingolipids. Sphingosine-1-phosphate is a ceramide derivative, acting antiapoptotically and mitogenically and it is importantly involved in cardioprotection. Further research on the involvement of sphingolipids in cellular pathophysiology may improve the prevention and therapy of lifestyle diseases.

## Introduction

Many experimental and clinical studies have described the role of sphingolipids in the pathogenesis of lifestyle diseases such as myocardial infarction, hypertension, stroke and diabetes mellitus. They are involved in the regulation of numerous cellular processes, including apoptosis [1]. The aim of this article is to summarize and present the role of selected sphingolipids in cardiovascular diseases and metabolic disorders.

## Review

### General characteristics of sphingolipids

**Fig. 1:**
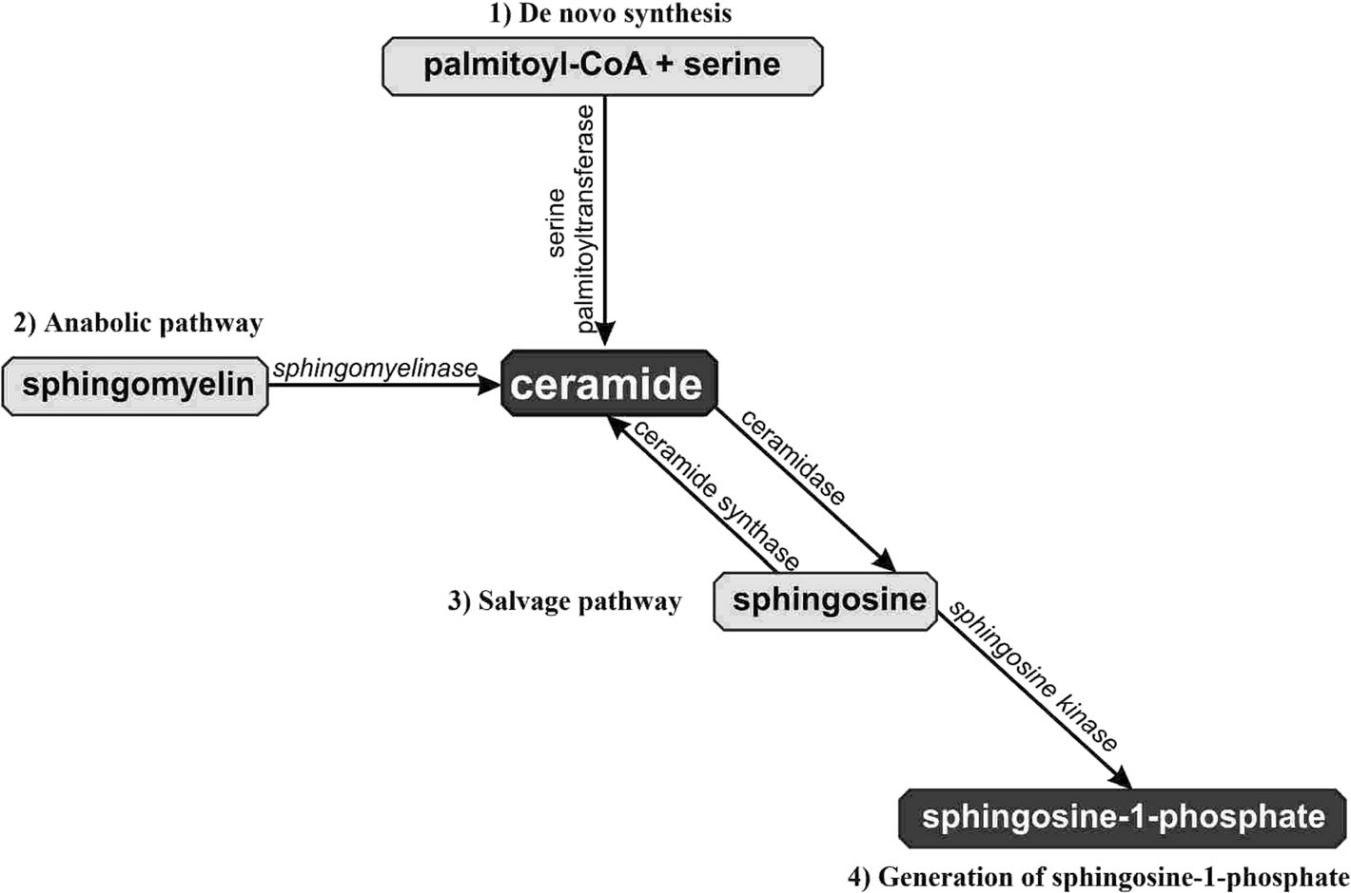
Pathways of ceramide generation

Sphingolipids – derivatives of amino alcohol sphingosine – are biologically active components of cell membranes. Sphingolipids play an important role in intracellular signal transduction and regulate cellular processes such as proliferation, maturation and apoptosis, and are also involved in cellular stress responses. One of the most important sphingolipids is ceramide (CER), which serves as a precursor for other biologically active sphingolipids, including sphingosine (SPH) and sphingosine-1-phosphate (S1P) [2, 3]. Many factors such as glucocorticosteroids, growth factors, interleukins, interferons, ionizing radiation and several chemotherapeutics induce cellular production of ceramide. There are three metabolic pathways involved in ceramide generation: 1) *de novo* synthesis in the cytosolic layer of endoplasmic reticulum via serine palmitoyltransferase (SPT); 2) hydrolysis of sphingomyelin via sphingomyelinase; and 3) production of ceramide from sphingosine via sphinganine N-acyltransferase (ceramide synthase) – salvage pathway (Fig. 1) [4–6]. *De novo* synthesis is the major source of ceramide in cells, although the hydrolysis of sphingomyelin via acid sphingomyelinase (aSMase) generates a large cellular amount of ceramide. There are three types of sphingomyelinases: magnesium-dependent acid sphingomyelinase, magnesium-independent neutral sphingomyelinase and alkaline sphingomyelinase. They differ in the optimum value of pH, molecular mass and reliance on divalent ions [4, 5]. Ceramidase catalyzes the hydrolysis of ceramide and leads to the production of sphingosine and a fatty acid. Sphingosine kinases (sphingosine kinase type 1; SK1 and sphingosine kinase type 2; SK2) may phosphorylate sphingosine to generate sphingosine-1-phosphate (S1P) [7].

As mentioned above, ceramide is the precursor of S1P, so any alteration of CER levels may increase the level of S1P. The main molecular mechanism of the functioning of sphingolipids is the activation of enzyme proteins, such as Ceramide-Activated Protein Kinase, Ceramide-Activated Protein Phosphatase, Mitogen Activated Protein Kinase, c-Jun-N-Terminal Protein Kinase [8]. CER acts as a second messenger, regulating many different processes such as cell growth, differentiation, senescence, necrosis, proliferation and apoptosis. Ceramide is also involved in regulation of protein kinase C, raf-1, kinase-suppressor of Ras, cellular protease cathepsin D and inhibition of phospholipase D [9, 10]. However, S1P plays a role in proliferation, cell growth, cell survival, cell migration, inflammation, angiogenesis, vasculogenesis and resistance to apoptotic cell death. The effect of S1P is mediated by the S1P receptors connected with different types of G-proteins, which results in activation of intracellular signaling pathways [9].

### Pathophysiological mechanisms of sphingolipids action

**Fig. 2:**
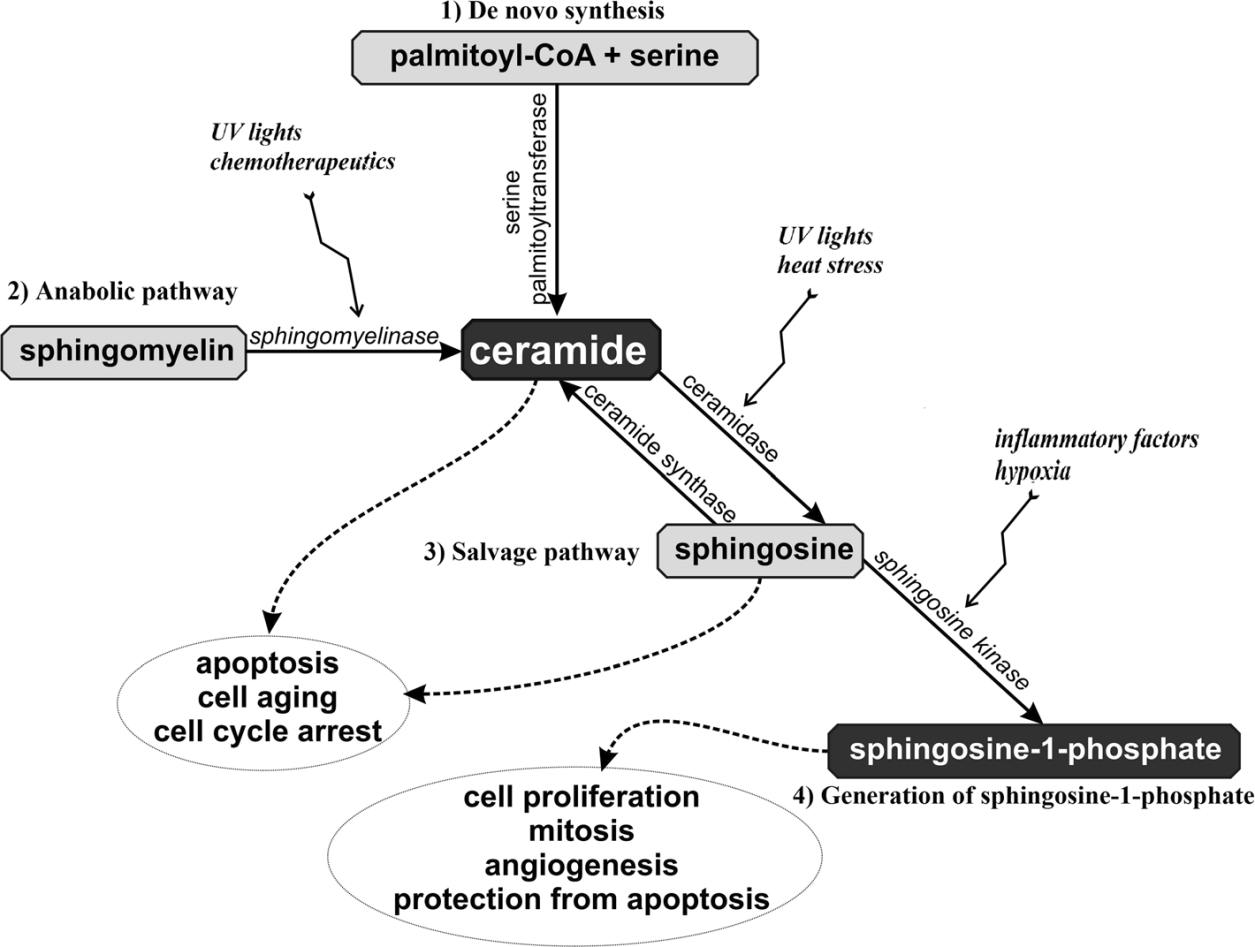
Pathophysiological mechanisms of sphingolipids action

Cellular apoptosis and stress responses are largely associated with ceramide. Many factors, including infection with, for example, Pseudomonas aeruginosa, Staphylococcus aureus, Neisseria gonorrhoeae, Rhinovirus, and also UV-light and some chemotherapeutics activate aSMase (Fig. 2). Some of these factors stimulate a translocation of aSMase to the outer leaflet of the cell membrane, leading to ceramide release and the formation of ceramide-enriched membrane domains. These domains serve to cluster membrane receptors to amplify signaling, which is required in the induction of apoptosis [11]. Many studies suggest that the relation between sphingolipids and apoptosis is important in the pathogenesis of many diseases, such as both diabetes mellitus type 1 and 2, stroke and myocardial infarction [6, 12, 13].

The aggregated lipoproteins isolated from human atherosclerotic lesions are enriched with ceramide. Moreover, an animal model of atherosclerosis suggested that treatment with myriocin, which is an inhibitor of SPT, may be associated with a protective lipoprotein profile. Although experimental observations suggest associations between sphingolipids, lipoproteins and atherosclerosis, the exact mechanisms still need to be determined [14].

The important role of sphingolipids in the pathogenesis of lifestyle diseases may be a result of their influence on the immune system. Experimental studies show the involvement of sphingolipids in trafficking and regulation of processes of immune cells such as T cell apoptosis, modification of Th1 vs Th2 T-cell balance, phagocytosis, inflammation and allergic excitability [15–20]. Some papers also suggest that inflammation may alter sphingolipid metabolism and S1P receptor activity [15, 21].

### Sphingolipids and other factors involved in cardiovascular diseases

The renin-angiotensin-aldosterone system plays an important role in vasoconstriction and salt and water retention. It can also cause, in some pathophysiological conditions, vascular hypertrophy and lead to hypertension. Ceramide seems to be responsible for some of these pathophysiological effects. *In vitro* studies performed on rat pheochromocytoma PC12W cells have shown that activation of angiotensin II type 2 receptors results in elevation of the intracellular CER level and apoptosis. Ceramide may also play a role in angiotensin II type 2 receptor-induced growth inhibition and apoptosis in cardiac and vascular tissues [22].

The increase in risk of cardiovascular and metabolic diseases has been described in some genetic disorders related to sphingolipid metabolism alterations. The abnormal glycosphingolipids accumulation in many types of tissues, which was observed in patients with Fabry disease probably was one of the causes of cardiovascular complications such as: valvular disease, coronary artery disease leading to myocardial infarction and also sudden cardiac death [23]. In Gaucher disease, in which lysosomal accumulation of glucocerebroside was presented, severe congestive cardiomyopathy and mitral and aortic valves disease were observed [24]. In lipid storage disorders such as Niemann-Pick and Sandhoff diseases and GM1 gangliosidosis, metabolic cardiomyopathy was also diagnosed [24].

### The role of sphingolipids in the cardiovascular system

Sphingolipids are components of the cardiomyocytes’ cell membrane. Some biologically active substances, for example, tumor necrosis factor-α (TNF-α) may induce synthesis of ceramide from sphingomyelin via sphingomyelinase. Ceramide, in turn, may act as a second messenger, promoting the apoptosis of cardiomyocytes [25–28]. On the other hand, sphingosine-1-phosphate is cardioprotective [27, 29, 30].

### Sphingolipids in ischemic heart disease

#### Experimental studies

Cordis et al. [31] observed a significant 50 % decrease in levels of sphingomyelin associated with significantly increased concentrations of ceramide during *in vitro* reperfusion of cardiomyocytes. Investigations performed in the animal model of myocardial ischemia/reperfusion demonstrated decreased activity of both acidic and neutral sphingomyelinase and significantly increased levels of ceramide [32].

#### Human studies

S1P serum level is a significant predictor of coronary artery disease (CAD) in patients undergoing coronary angiography [33]. Egom et al. [34] reported significantly increased plasma levels of S1P and SPH in patients 1 and 5 min after percutaneous coronary intervention, most probably resulting from transient ischemia.

### Sphingolipids – myocardial infarction

#### Experimental studies

*In vitro* experiments performed on isolated murine hearts treated with ischemic preconditioning (IPC) revealed significantly increased activity of SK, an elevated level of S1P and a reduction of the infarction area. Dimethylsphingosine, an inhibitor of SK, significantly decreased the cardioprotective effect of IPC [35].

Experimental myocardial infarction induced in male Wistar rats showed significant alterations of sphingolipid levels in plasma, erythrocytes and platelets after ligation of the left coronary artery. The plasma levels of S1P significantly decreased, but the plasma levels of CER significantly increased at 1 and 6 h after myocardial infarction, but both sphingolipids returned to the control values after 24 h from the cardiac incident [27]. Knapp et al. [12] observed, in the uninfarcted area of the left ventricle, a significant triple reduction in the S1P level at 1 and 6 h after incident and a further reduction after 24 h. The ceramide level was significantly decreased 6 h after myocardial infarction. The authors suggested that alteration of the S1P/CER ratio may be responsible for apoptosis of the cardiomyocytes from the uninfarcted area of the myocardium.

#### Human studies

Analysis of patients with CAD has shown that plasma ceramide levels and SMase activity were elevated in the stable angina pectoris (SAP), unstable angina pectoris (UAP) and acute myocardial infarction (AMI) groups. In the group of patients with UAP, there was a significant increase in SMase activity and ceramide concentration in comparison to the control and SAP groups, although the increased activity of SMase was transient. In the AMI group, the significantly elevated level of ceramide was noted up to 7 days following the cardiac incident in comparison to the control and SAP groups, whereas enhancement in activity of SMase occured only 3 days after infarction. The authors of this study suggest that higher ceramide levels and SMase activity in patients with coronary heart disease may be an important factor in the development of atherosclerosis and changes in the plasma concentration of sphingolipids may indicate their involvement in the molecular mechanism of plaque destabilization [36].

The plasma concentration of CER in patients with acute myocardial infarction after admission to the intensive heart care unit was not significantly changed when compared to the control group, whereas the concentration of S1P was decreased by a significant 50 % [37]. Another investigation revealed that the plasma level of S1P was significantly decreased in patients admitted to hospital with STEMI (ST-elevation myocardial infarction) and ceramide level was reduced 5 days post-infarct compared to the control group, although the reduction in ceramide level reached statistical significance 30 days following infarction. Two years after the infarction the plasma S1P level almost completely recovered, whereas the decreased ceramide level was maintained. Erythrocytes from STEMI patients showed accumulation of S1P and ceramide during the thirty days following infarction, although only the elevated level of S1P reached statistical significance during the whole time of observation. After two years of observation the concentration of CER and S1P decreased to the control levels, although these findings were not statistically significant [38].

### Sphingolipids in hypertension

#### Experimental studies

*In vitro* experiments performed on carotid arteries isolated from spontaneously hypertensive rats (SHR) demonstrated significant contraction of vessels after the application of sphingomyelinase or SK inhibitors. This effect was not observed in arteries isolated from normotensive WKY rats [39]. Presumably, ceramide, which causes elevated thromboxane A2 release from the endothelium, contributed to the contraction of the isolated arteries.

S1P triggers both vasoconstriction and vasodilation, depending on the type of activated receptor. S1P triggers endothelial nitric oxide synthase-dependent vasodilation in epinephrine preconstricted mesenteric arterioles from either rats or mice. Many studies have proven that the activation of nitric oxide synthase may be mediated by the S1P_1_ receptor. S1P induces vasoconstriction in basilar arteries from wild type and S1P_2_ knockout mice, which presumably results from the activation of S1P_3_ subtype. In addition, the vasoconstricting activity of S1P was lost in S1P_3_ knockout animals [40].

Fryer et al. [41] observed dose-dependent and sustained elevation in mean arterial blood pressure in Sprague Dawley rats resulting from oral administration of unselective S1P receptor agonist (FTY720). Four weeks of treatment with losartan or hydralazine significantly decreased blood pressure and vascular CER level, with no concomitant reduction of plasma ceramide concentration, suggesting that only vascular CER level is sensitive to antihypertensive therapy [42]. The exact mechanism, in which falling blood pressure leads to the reduction of vascular ceramide levels is currently unknown. Although stimulation of angiotensin II receptor type 2 increases cellular ceramide levels, it does not explain the interaction between losartan, an antagonist of angiotensin II type 1 receptor, and vascular levels of ceramide [22]. A mechanism, which may explain the observed effect of both hydralazine and losartan on the vascular ceramide level, has been proposed by Czarny et al. The authors suggested that neutral SMase in plasma membrane acts as a mechanosensor, whose activity may be enhanced by high shear stress, which results in the generation of ceramide [43]. The link between hypertension and ceramides suggests a novel pathophysiological mechanism leading to endothelial dysfunction and abnormal blood pressure regulation, which needs to be determined. This mechanism can also suggest the target for new drugs modulating the sphingolipid system and metabolism to improve the pharmacological treatment of hypertension [42].

#### Human studies

Spijkers et al. reported that the ceramide level is increased in patients with hypertension and that the concentration of ceramide correlates with the severity of disease [39].

### Sphingolipids in stroke

#### Experimental studies

*In vitro* experiments on neuroblastoma cells indicated that an elevated ceramide level in neurons during ischemia/reperfusion was responsible for apoptosis, however, a low level was a factor for the survival of the neurons [5, 13, 44].

Sphingolipids are presumably involved in the pathogenesis of stroke. Nakane et al. [45] observed a significant increase in ceramide level and also a significant decrease in sphingomyelin level in the hippocampus of the gerbil after 30 min and 24 h following 5 min of ischemia. Middle cerebral artery occlusion (MCAO) induced an elevation of ceramide concentration in the rat cerebral cortex [46, 47]. In SHR rats, transient ischemia performed prior to the MCAO (preconditioning) significantly reduced ceramide levels both in ischemic and perifocal areas [47]. In the mouse model of ischemic stroke, attenuation of SMase activity significantly reduced brain tissue injury [48, 49].

Sphingosine-1-phosphate, contrary to ceramide, is neuroprotective during ischemia [50, 51]. In mice with middle cerebral artery thrombosis, the level of S1P significantly decreased 3 days after ischemic stroke and then progressively increased, reaching maximum concentration 14 days after occlusion [51]. Peritoneal application of S1P agonist (FTY720) activated S1P1 receptors in Sprague Dawley rats with MCAO and significantly reduced the area of damaged brain tissue [52]. Presumably S1P regulates prosurvival mechanisms through suppression of proapoptotic factors, including caspase 3, and activation of protein kinase B (Akt) and extracellular regulated-signal kinases (ERKs), both involved in cell survival [52].

#### Human studies

Kubota et al. reported that the neural ceramide level was increased in patients with an acute case of internal carotid artery occlusion. Presumably, this elevation resulted from excessive degradation of ganglioside-sphingolipids, which are present in high concentration in the central nervous system. In cells, gangliosides are localized in the outer leaflets of plasma membranes and cell surface microdomains, participating in cell-cell recognition, adhesion and signal transduction [53, 54].

### Sphingolipids in diabetes mellitus

#### Experimental studies

Many *in vitro* investigations confirm the contribution of sphingolipids to the pathogenesis of diabetes mellitus type 1 and 2. Incubation of β cells isolated from wild type mice in ceramide induced apoptosis [55]. Isolated murine β cells lacking caspase 8 – a crucial substance in death receptor-mediated apoptosis, incubated in ceramide, showed increased viability in comparison to the control β cells, suggesting that ceramide is importantly involved in β cell apoptosis [56].

Maestre et al. [57] observed that in the presence of fatty acids (for example, palmitic acid – a substrate for *de novo* synthesis of ceramide) INS-1 cells (a line of pancreatic β cells) demonstrated increased permeability of the mitochondrial membrane to cytochrome c and Apoptosis Inducing Factor. Moreover, the concentration of proapoptotic protein Bax was also elevated. Birbes et al. [58] studied the involvement of ceramide in the increase of mitochondrial membrane permeability to cytochrome c using proapoptotic protein Bax in breast cancer cells MCF7. Ceramide presumably induces apoptosis through disruption of electron transport in complex I and III of the mitochondrial respiratory chain and activation of the mitochondrial NADPH oxidase, which contributes to increased synthesis of the Reactive Oxygen Species [6, 59, 60].

*In vitro* and *in vivo* experiments suggest the role of ceramide in insulin resistance [61–64]. Ceramide significantly decreased the expression of insulin mRNA in pancreatic β cells isolated from Wistar rats [65]. Interestingly, in mice, the genetic deficiency of ceramide kinase (CERK), phosphorylating CER to ceramide-1-phosphate significantly improved the insulin sensitivity [61]. Intravenous infusion of LDL (Low Density Lipoprotein) – ceramide caused insulin resistance in lean mice with concomitant reduction in skeletal muscles’ glucose uptake [62].

Oral administration of S1P receptor agonist (FTY720) significantly increased the mass of pancreatic β cells, significantly increased the concentration of insulin in blood and the normalized blood glucose level, with no effect on the cellular insulin sensitivity in db/db mice with genetically determined diabetes mellitus type 2 [66]. Sphingosine kinase knockout mice fed on a high-fat diet presented diabetes, simultaneous significant reduction of pancreatic β cell mass and plasma insulin level, whereas wild type mice developed glucose intolerance, significantly increased mass of pancreatic β cells and hyperinsulinemia [67].

Sphingolipids are also involved in the development of diabetic nephropathy. Studies performed on mesangial cells isolated from the kidney of diabetic rats have proven that S1P induced mesangial cell proliferation both under normoglycemic and hyperglycemic conditions, and enhanced the expression of fibronectin [68].

#### Human studies

The significant elevation of plasma ceramide levels was observed in patients with diabetes mellitus [62, 63]. The Roux-en-Y bypass gastric surgery significantly decreased the plasma ceramide level in severely obese patients. After 6 months post operation, the reduction in total ceramide level was also significantly correlated with excess weight loss, improvement in insulin sensitivity and decrease in TNF α concentration [64]. The authors suggest that a reduced inflammatory environment resulting from the loss of adipose tissue causing lipotoxicity and cellular dysfunction may explain the observed improvement in insulin sensitivity.

Incubation of human pancreatic β cells with S1P receptor agonist did not have a negative influence on the insulin secretion triggered by glucose administration and did not induce apoptosis [69].

A decreased plasma level of very long chain ceramides in patients with diabetes type 1 was correlated with progression to macroalbuminuria in comparison to diabetic patients with normoalbuminuria [70]. The authors of the study suggest that ceramides play a regulatory role in pathways, which may lead to a loss of renal function. Ceramides are an integral part of cell membrane structure and they act as signaling molecules. The content of ceramides in kidney cell membranes may be changed by interaction with plasma lipoproteins containing altered ceramide compositions. Changes in cell-lipoprotein interactions may trigger the cell’s signaling pathways altering intracellular sphingolipid metabolism. Many investigations suggest that ceramides are also involved strongly in the pathogenesis of insulin resistance, which together with other metabolic syndrome elements are known to be risk factors of diabetes complications and macrovascular disease in patients with and without type 2 diabetes. Mechanisms leading to the alteration of kidney cell membranes’ ceramide composition and association between plasma lipoproteins and kidney cell sphingolipid metabolism and its pathophysiological implications in patients with diabetes mellitus and albuminuria are yet to be determined [70].

## Conclusions

Sphingolipids are supposed to play an important role in the regulation of many cellular functions. Many studies suggest that interactions between two main sphingolipids, ceramide and sphingosine-1-phosphate are essential for the appropriate functioning of the human body. Any disturbances in the balanced relationship between those two sphingolipids accelerates the induction of apoptosis, a process involved in the pathogenesis of diseases such as myocardial infarction, stroke and diabetes mellitus type 2. Pharmacological modification of sphingolipid metabolism may be very important in the treatment and prevention of lifestyle diseases. It seems that, for example, inhibition of *de novo* ceramide synthesis may be beneficial to improve myocardial systolic function in ischemic heart disease, but attenuation of CERK can improve the insulin sensitivity.

## Abbreviations

Akt: Potein kinase B
aSMase: Acid sphingomyelinase
CAD: Coronary artery disease
CER: Ceramide
CERK: Ceramide kinase
ERK: Extracellular regulated-signal kinase
FTY720: S1P receptor agonist
IPC: Ischemic preconditioning
LDL: Low density lipoprotein
MCAO: Middle cerebral artery occlusion
SHR: Spontaneously hypertensive rats
SK1: Sphingosine kinase type 1
SK2: Sphingosine kinase type 2
S1P: Sphingosine-1-phosphate
SPH: Sphingosine
SPT: Serine palmitoyltransferase
STEMI: (ST-elevation myocardial infarction)
TNF-α: Tumor necrosis factor-α.

## References

[1] Tirodkar TS, Voelkel-Johnson C (2012). Sphingolipids in apoptosis. Exp Oncol.

[2] Mao C, Obeid LM (2008). Ceramidases: regulators of cellular responses mediated by ceramide, sphingosine, and sphingosine-1-phosphate. Biochim Biophys Acta.

[3] Paugh SW, Paugh BS, Rahmani M, Kapitonov D, Almenara JA, Kordula T (2008). A selective sphingosine kinase 1 inhibitor integrates multiple molecular therapeutic targets in human leukemia. Blood.

[4] Gulbins E, Li PL (2006). Physiological and pathophysiological aspects of ceramide. Am J Physiol Regul Integr Comp Physiol.

[5] Car H, Żendzian-Piotrowska M, Fiedorowicz A, Prokopiuk S, Sadowska A, Kurek K (2012). The role of ceramides in selected brain pathologies: ischemia/hypoxia, Alzheimer disease. Postepy Hig Med Dosw (Online).

[6] Galadari S, Rahman A, Pallichankandy S, Galadari A, Thayyullathil F (2013). Role of ceramide in diabetes mellitus: evidence and mechanisms. Lipids Health Dis.

[7] Ponnusamy S, Meyers-Needham M, Senkal CE, Saddoughi SA, Sentelle D, Selvam SP (2010). Sphingolipids and cancer: ceramide and sphingosine-1-phosphate in the regulation of cell death and drug resistance. Future Oncol.

[8] Kurek K, Piotrowska DM, Wiesiołek-Kurek P, Chabowska A, Łukaszuk B, Żendzian-Piotrowska M (2013). The role of sphingolipids in selected cardiovascular diseases. Postepy Hig Med Dosw (Online).

[9] Bartke N, Hannun YA (2009). Bioactive sphingolipids: metabolism and function. J Lipid Res.

[10] Gangoiti P, Camacho L, Arana L, Ouro A, Granado MH, Brizuela L, Casas J (2010). Control of metabolism and signaling of simple bioactive sphingolipids: Implications in disease. Prog Lipid Res.

[11] Schenck M, Carpinteiro A, Grassmé H, Lang F, Gulbins E (2007). Ceramide: physiological and pathophysiological aspects. Arch Biochem Biophys.

[12] Knapp M, Zendzian-Piotrowska M, Kurek K, Błachnio-Zabielska A (2012). Myocardial infarction changes sphingolipid metabolism in the uninfarcted ventricular wall of the rat. Lipids.

[13] Herr I, Martin-Villalba A, Kurz E, Roncaioli P, Schenkel J, Cifone MG (1999). FK506 prevents stroke-induced generation of ceramide and apoptosis signaling. Brain Res.

[14] Tabas I (2004). Sphingolipids and atherosclerosis: a mechanistic connection? A therapeutic opportunity?. Circulation.

[15] Yang J, Yu Y, Sun S, Duerksen-Hughes PJ (2004). Ceramide and other sphingolipids in cellular responses. Cell Biochem Biophys.

[16] Cuvillier O, Edsall L, Spiegel S (2000). Involvement of sphingosine in mitochondria-dependent Fas-induced apoptosis of type II Jurkat T cells. J Biol Chem.

[17] Tokura Y, Wakita H, Yagi H, Nishimura K, Furukawa F, Takigawa M (1996). Th2 suppressor cells are more susceptible to sphingosine than Th1 cells in murine contact photosensitivity. J Invest Dermatol.

[18] Suchard SJ, Hinkovska-Galcheva V, Mansfield PJ, Boxer LA, Shayman JA (1997). Ceramide inhibits IgG-dependent phagocytosis in human polymorphonuclear leukocytes. Blood.

[19] Lamour NF, Subramanian P, Wijesinghe DS, Stahelin RV, Bonventre JV, Chalfant CE (2009). Ceramide 1-phosphate is required for the translocation of group IVA cytosolic phospholipase A2 and prostaglandin synthesis. J Biol Chem.

[20] Prieschl EE (1999). The balance between sphingosine and sphingosine-1-phosphate is decisive for mast cell activation after Fc epsilon receptor I triggering. J Exp Med.

[21] Maceyka M, Spiegel S (2014). Sphingolipid metabolites in inflammatory disease. Nature.

[22] Berry C, Touyz R, Dominiczak AF, Webb RC, Johns DG (2001). Angiotensin receptors: signaling, vascular pathophysiology, and interactions with ceramide. Am J Physiol Heart Circ Physiol.

[23] Zarate YA, Hopkin RJ (2008). Fabry’s disease. Lancet.

[24] Guertl B, Noehammer C, Hoefler G (2000). Metabolic cardiomyopathies. Int J Exp Pathol.

[25] Tepper CG, Jayadev S, Liu B, Bielawska A, Wolff R, Yonehara S (1995). Role for ceramide as an endogenous mediator of Fas-induced cytotoxicity. Proc Natl Acad Sci U S A.

[26] Testi R (1996). Sphingomyelin breakdown and cell fate. Trends Biochem Sci.

[27] Knapp M, Zendzian-Piotrowska M, Błachnio-Zabielska A, Zabielski P, Kurek K, Górski J (2012). Myocardial infarction differentially alters sphingolipid levels in plasma, erythrocytes and platelets of the rat. Basic Res Cardiol.

[28] Parra V, Moraga F, Kuzmicic J, López-Crisosto C, Troncoso R, Torrealba N (1832). Calcium and mitochondrial metabolism in ceramide-induced cardiomyocyte death. Biochim Biophys Acta.

[29] Jin ZQ, Zhou HZ, Zhu P, Honbo N, Mochly-Rosen D, Messing RO (2002). Cardioprotection mediated by sphingosine-1-phosphate and ganglioside GM-1 in wild-type and PKC epsilon knockout mouse hearts. Am J Physiol Heart Circ Physiol.

[30] Karliner JS (1831). Sphingosine kinase and sphingosine 1-phosphate in the heart: a decade of progress. Biochim Biophys Acta.

[31] Cordis GA, Yoshida T, Das DK (1998). HPTLC analysis of sphingomyelin, ceramide and sphingosine in ischemic/reperfused rat heart. J Pharm Biomed Anal.

[32] Zhang DX, Fryer RM, Hsu AK, Zou AP, Gross GJ, Campbell WB (2001). Production and metabolism of ceramide in normal and ischemic-reperfused myocardium of rats. Basic Res Cardiol.

[33] Deutschman DH, Carstens JS, Klepper RL, Smith WS, Page MT, Young TR (2003). Predicting obstructive coronary artery disease with serum sphingosine-1-phosphate. Am Heart J.

[34] Egom EE, Mamas MA, Chacko S, Stringer SE, Charlton-Menys V, El-Omar M (2013). Serum sphingolipids level as a novel potential marker for early detection of human myocardial ischaemic injury. Front Physiol.

[35] Jin ZQ, Goetzl EJ, Karliner JS (2004). Sphingosine kinase activation mediates ischemic preconditioning in murine heart. Circulation.

[36] Pan W, Yu J, Shi R, Yan L, Yang T, Li Y (2014). Elevation of ceramide and activation of secretory acid sphingomyelinase in patients with acute coronary syndromes. Coron Artery Dis.

[37] Knapp M, Baranowski M, Czarnowski D, Lisowska A, Zabielski P, Górski J (2009). Plasma sphingosine-1-phosphate concentration is reduced in patients with myocardial infarction. Med Sci Monit.

[38] Knapp M, Lisowska A, Zabielski P, Musiał W, Baranowski M (2013). Sustained decrease in plasma sphingosine-1-phosphate concentration and its accumulation in blood cells in acute myocardial infarction. Prostaglandins Other Lipid Mediat.

[39] Spijkers LJA, van den Akker RFP, Janssen BJA, Debets JJ, De Mey JG, Stroes ES (2011). Hypertension Is Associated with Marked Alterations in Sphingolipid Biology: A Potential Role for Ceramide. PLoS ONE.

[40] Igarashi J, Michel T (2009). Sphingosine-1-phosphate and modulation of vascular tone. Cardiovasc Res.

[41] Fryer RM, Muthukumarana A, Harrison PC, Nodop Mazurek S, Chen RR, Harrington KE (2012). The clinically-tested S1P receptor agonists, FTY720 and BAF312, demonstrate subtype-specific bradycardia (S1P_1_) and hypertension (S1P_3_) in rat. PLoS ONE.

[42] Spijkers LJA, Janssen BJA, Nelissen J, Meens MJ, Wijesinghe D, Chalfant CE (2011). Antihypertensive treatment differentially affects vascular sphingolipid biology in spontaneously hypertensive rats. PLoS ONE.

[43] Czarny M, Schnitzer JE (2004). Neutral sphingomyelinase inhibitor scyphostatin prevents and ceramide mimics mechanotransduction in vascular endothelium. Am J Physiol Heart Circ Physiol.

[44] Agudo-López A, Miguel BG, Fernández I, Martínez AM (2011). Role of protein kinase C and mitochondrial permeability transition pore in the neuroprotective effect of ceramide in ischemia-induced cell death. FEBS Lett.

[45] Nakane M, Kubota M, Nakagomi T, Tamura A, Hisaki H, Shimasaki H (2000). Lethal fore-brain ischemia stimulates sphingomyelin hydrolysis and ceramide generation in the gerbil hippocampus. Neurosci Lett.

[46] Kubota M, Narita K, Nakagomi T, Tamura A, Shimasaki H, Ueta N (1996). Sphingomyelin changes in rat cerebral cortex during focal ischemia. Neurol Res.

[47] Takahashi K, Ginis I, Nishioka R, Klimanis D, Barone FC, White RF (2004). Glucosylceramide synthase activity and ceramide levels are modulated during cerebral ischemia after ischemic preconditioning. J Cereb Blood Flow Metab.

[48] Yu ZF, Nikolova-Karakashian M, Zhou D, Cheng G, Schuchman EH, Mattson MP (2000). Pivotal role for acidic sphingomyelinase in cerebral ischemia-induced ceramide and cytokine production, and neuronal apoptosis. J Mol Neurosci.

[49] Soeda S, Tsuji Y, Ochiai T, Mishima K, Iwasaki K, Fujiwara M (2004). Inhibition of sphingomyelinase activity helps to prevent neuron death caused by ischemic stress. Neurochem Int.

[50] Kimura A, Ohmori T, Ohkawa R, Madoiwa S, Mimuro J, Murakami T (2007). Essential roles of sphingosine 1-phosphate/S1P1 receptor axis in the migration of neural stem cells toward a site of spinal cord injury. Stem Cells.

[51] Kimura A, Ohmori T, Kashiwakura Y, Ohkawa R, Madoiwa S, Mimuro J (2008). Antagonism of sphingosine 1-phosphate receptor-2 enhances migration of neural progenitor cells toward an area of brain. Stroke.

[52] Hasegawa Y, Suzuki H, Sozen T, Rolland W, Zhang JH (2010). Activation of sphingosine 1-phosphate receptor-1 by FTY720 is neuroprotective after ischemic stroke in rats. Stroke.

[53] Kubota M, Kitahara S, Shimasaki H, Ueta N (1989). Accumulation of ceramide in ischemic human brain of an acute case of cerebral occlusion. Jpn J Exp Med.

[54] Yu RK, Tsai YT, Ariga T, Yanagisawa M (2011). Structures, biosynthesis, and functions of gangliosides - an overview. J Oleo Sci.

[55] Zhang Y, Ranta F, Tang C, Shumilina E, Mahmud H, Föller M (2009). Sphingomyelinase dependent apoptosis following treatment of pancreatic beta-cells with amyloid peptides Abeta(1-42) or IAPP. Apoptosis.

[56] Liadis N, Salmena L, Kwan E, Tajmir P, Schroer SA, Radziszewska A (2007). Distinct *in vivo* roles of caspase-8 in beta-cells in physiological and diabetes models. Diabetes.

[57] Maestre I, Jordan J, Calvo S, Reig JA, Ceña V, Soria B (2003). Mitochondrial dysfunction is involved in apoptosis induced by serum withdrawal and fatty acids in the beta-cell line INS-1. Endocrinology.

[58] Birbes H, Luberto C, Hsu YT, El Bawab S, Hannun YA, Obeid LM (2005). A mitochondrial pool of sphingomyelin is involved in TNFalpha-induced Bax translocation to mitochondria. Biochem J.

[59] Di Paola M, Cocco T, Lorusso M (2000). Ceramide interaction with the respiratory chain of heart mitochondria. Biochemistry.

[60] Gudz TI, Tserng KY, Hoppel CL (1997). Direct inhibition of mitochondrial respiratory chain complex III by cell-permeable ceramide. J Biol Chem.

[61] Mitsutake S, Date T, Yokota H, Sugiura M, Kohama T, Igarashi Y (2012). Ceramide kinase deficiency improves diet-induced obesity and insulin resistance. FEBS Lett.

[62] Boon J, Hoy AJ, Stark R, Brown RD, Meex RC, Henstridge DC (2013). Ceramides contained in LDL are elevated in type 2 diabetes and promote inflammation and skeletal muscle insulin resistance. Diabetes.

[63] Lopez X, Goldfine AB, Holland WL, Gordillo R, Scherer PE (2013). Plasma ceramides are elevated in female children and adolescents with type 2 diabetes. J Pediatr Endocrinol Metab.

[64] Huang H, Kasumov T, Gatmaitan P (2011). Gastric bypass surgery reduces plasma ceramide subspecies and improves insulin sensitivity in severely obese patients. Obesity (Silver Spring).

[65] Kelpe CL, Moore PC, Parazzoli SD, Wicksteed B, Rhodes CJ, Poitout V (2003). Palmitate inhibition of insulin gene expression is mediated at the transcriptional level via ceramide synthesis. J Biol Chem.

[66] Zhao Z, Choi J, Zhao C, Ma ZA (2012). FTY720 normalizes hyperglycemia by stimulating β-cell *in vivo* regeneration in db/db mice through regulation of cyclin D3 and p57(KIP2). J Biol Chem.

[67] Qi Y, Chen J, Lay A, Don A, Vadas M, Xia P (2013). Loss of sphingosine kinase 1 predisposes to the onset of diabetes via promoting pancreatic β-cell death in diet-induced obese mice. FASEB J.

[68] Liu W, Lan T, Xie X, Huang K, Peng J, Huang J (2012). S1P2 receptor mediates sphingosine-1-phosphate-induced fibronectin expression via MAPK signaling pathway in mesangial cells under high glucose condition. Exp Cell Res.

[69] Truong W, Emamaullee JA, Merani S, Anderson CC, James Shapiro AM (2007). Human islet function is not impaired by the sphingosine-1-phosphate receptor modulator FTY720. Am J Transplant.

[70] Klein RL, Hammad SM, Baker NL, Hunt KJ, Al Gadban MM, Cleary PA (2014). Decreased plasma levels of select very long chain ceramide species are associated with the development of nephropathy in type 1 diabetes. Metabolism.

